# Effect of advanced glycation end products on nocturia or sleep disorders: A longitudinal study

**DOI:** 10.1002/bco2.114

**Published:** 2021-10-05

**Authors:** Sakae Konishi, Shingo Hatakeyama, Atsushi Imai, Kazutaka Okita, Koichi Kido, Yusuke Ozaki, Nozomi Uemura, Takuro Iwane, Teppei Okamoto, Hayato Yamamoto, Takahiro Yoneyama, Yasuhiro Hashimoto, Chikara Ohyama

**Affiliations:** ^1^ Department of Urology Hirosaki University Graduate School of Medicine Hirosaki Japan; ^2^ Department of Urology Oyokyo Kidney Research Institute Hirosaki Japan; ^3^ Innovation Center for Health Promotion Hirosaki University Graduate School of Medicine Hirosaki Japan; ^4^ Hirosaki University COI Research Initiative Organization Hirosaki University Graduate School of Medicine Hirosaki Japan; ^5^ Department of Advanced Transplant and Regenerative Medicine Hirosaki University Graduate School of Medicine Hirosaki Japan

**Keywords:** advanced glycation end products, longitudinal, nocturia, oxidative stress, sleep disorders

## Abstract

**Objective:**

This study aimed to investigate the effect of advanced glycation end products (AGEs) on nocturia and sleep disorders in community‐dwelling adults.

**Materials and Methods:**

This longitudinal study evaluated AGEs level, sleep disorders and nocturia frequency in 447 community‐dwelling adults between May 2011 and May 2016. Sleep disorders were evaluated using the Pittsburgh Sleep Quality Index (PSQI) score. Participants were divided into two groups: AGEs‐low (≤1.80) and AGEs‐high (>1.80). The longitudinal nocturia and PSQI changes for 5 years between the AGEs‐low and AGEs‐high groups were compared. The effect of baseline oxidative stress on worsening of nocturia or PSQI score 5 years later was evaluated using multivariable logistic regression analyses.

**Results:**

There was no significant difference in PSQI score and nocturia frequency between the AGEs‐low (*n* = 223) and AGEs‐high (*n* = 224) groups at baseline. The PSQI score and nocturia frequency increased significantly over 5 years in both groups. However, the PSQI score increased significantly in the AGEs‐high group compared with the AGEs‐low group, although there was no difference in nocturia. Background‐adjusted multivariable analysis showed that the AGE‐high was significantly associated with PSQI score worsening, but AGE‐high was not significantly associated with nocturia worsening.

**Conclusions:**

Oxidative stress may be significantly associated with worsening sleep disorders, although oxidative stress may not significantly worsen nocturia frequency.

## 1 INTRODUCTION

Nocturia is a complaint wherein an affected person must awaken during the main sleep period to urinate.^1^, ^2^, ^3^, ^4^ Nocturia was defined by The International Continence Society as ‘the number of times urine is passed during the main sleep period. Having woken to pass urine for the first time, each urination must be followed by sleep or the intention to sleep. This should be quantified using a bladder diary’.^5^ It remarkably reduces health status and quality of life in older individuals.^6^, ^7^, ^8^, ^9^ Consequently, sleep disorders have a close relationship with nocturia due to frequent nocturnal awakening.^10^, ^11^, ^12^ Recent longitudinal studies suggested a negative influence of sleep problems on nocturia.^13^, ^14^ However, the exact association of nocturia with sleep disorders is difficult to define due to complexity.^15^

Oxidative stress is an imbalance between free radicals and antioxidants and has been defined as harmful because oxygen free radicals attack biological molecules such as lipids, proteins and DNA.^16^, ^17^ A previous cross‐sectional study suggested a significant association of oxidative stress with nocturia.^17^ Also, the relationship between oxidative stress and sleep disorders were reported in obstructive sleep apnoea syndrome (OSAS)/irritable bowel syndrome.^18^, ^19^, ^20^ However, no study has addressed the association between oxidative stress and sleep disorders in the general population. In addition, no study is available on the relationship between oxidative stress, sleep disorders and nocturia.

Advanced glycation end products (AGEs), measured by the skin autofluorescence levels of the forearm, are one of the surrogate markers of oxidative stress.^21^ AGEs include a group of harmful compounds, modified proteins and/or lipids with damaging potential. Skin AGEs increased in patients with diabetes, renal failure, vascular complications and coronary heart disease.^21^, ^22^ The current study hypothesized that the effect of oxidative stress might have a key role in worsening of nocturia and/or sleep disorder.^17^

This longitudinal study aimed to investigate the effect of oxidative stress on sleep disorder or nocturia in community‐dwelling adults for 5 years.

## 2 MATERIALS AND METHODS

### 2.1 Design and ethics statement

The study complied with the ethical standards of the Declaration of Helsinki. The Ethics Committee of Hirosaki University School of Medicine approved the study. All subjects provided written informed consent before participating in the study. This observational study was registered at the UMIN‐CTR (UMIN000039744).

### 2.2 Participant selection, data collection and evaluation

The Iwaki Health Promotion Project was a comprehensive study clarifying the aetiology of lifestyle‐related disease to promote health and extend the lifespan of residents of the city of Hirosaki (Iwaki district) in the northern part of Japan.^14^, ^23^, ^24^ Of those, sleep disorders, nocturia frequency and oxidative stress in 521 adults in the Iwaki Health Promotion Project were longitudinally evaluated with baseline and 5‐year follow‐up between 2011 and 2016. Information on the baseline characteristics (age, gender, body mass index and smoking history), history of hypertension (HTN), cardiovascular disease (CVD), and type 2 diabetes mellitus (DM) was obtained via a questionnaire. The somatic fat rate was measured using a multifrequency bioelectrical impedance analyser (InBody 770, InBody Japan, Tokyo). HTN was defined as a systolic and diastolic blood pressure of 140 or more and 90 mmHg or more, respectively, or taking antihypertensive medication. Diabetic participants were those with a history of type 2 DM or those who met the relevant diagnostic criteria and required glycaemic control. CVD was a positive history of cardiac surgery, myocardial infarction, angina, stroke or taking any cardiotonic agents. Sleep disorder was measured using the Japanese version of the Pittsburgh Sleep Quality Index (PSQI).^25^ The total scores assessed overall sleep quality (range, 0–21), and sleep disorders were defined as a PSQI > 5. Frequency of nocturia was measured using the International Prostate Symptom Score.

### 2.3 Oxidative stress evaluation

AGEs are sugar‐modified adducts that accumulate in the body as one of the ageing substances. An Advanced Glycation End Products Reader (AGE Reader; Diagnostics Technologies B.V., Netherlands) can quantitatively evaluate oxidative stress using ultra‐violet light to excite autofluorescence in human skin tissue. The AGE Reader is a non‐invasive method that measures AGEs accumulation in the skin by placing our forearm in 12 s. Skin autofluorescence is calculated as the ratio of the mean intensities detected from the skin between 420–600 and 300–420 nm. It correlates with collagen, protein and lipid‐linked fluorescence and specific to the systematic accumulation of AGEs in the body as a long‐term tissue damage.

### 2.4 Outcomes

Participants were divided into two groups according to the median value of skin AGEs: AGEs‐low and AGEs‐high. The longitudinal change of nocturia and PSQI for 5 years between the AGEs‐low and AGE‐high groups were compared. The effect of baseline AGEs on worsening of nocturia or PSQI score after 5‐year follow‐up was evaluated using multivariable logistic regression analyses.

### 2.5 Statistical analysis

Statistical analysis was performed with GraphPad Prism ver. 7.00 (GraphPad Software, San Diego, CA, USA), Microsoft Excel (Microsoft Corporation, Redmond, WA, USA), BellCurve for Excel (Social Survey Research Information Co., Ltd., Tokyo, Japan). Categorical variables were compared using Fisher's exact test or the *χ* ^2^ test. Quantitative variables were expressed as the median and interquartile range (IQR). The differences between groups were compared using the Student's *t* test for normally distributed data or the Mann–Whitney *U* test for non‐normally distributed data. A multivariable logistic regression analysis investigated the effect of baseline AGEs on PSQI or nocturia worsening. The analysis used the inverse probability of a treatment weighting (IPTW) model.^26^, ^27^ The odds ratio (OR) with 95% confidence interval (CI) was calculated after controlling for potential confounders at baseline, including patient age, gender, body mass index, somatic fat rate, HTN, CVD, DM, smoking history, sleeping tablet use, nocturia and PSQI. Differences were considered statistically significant at *P* < 0.05.

## 3 RESULTS

### 3.1 Baseline characteristics

**FIGURE 1:**
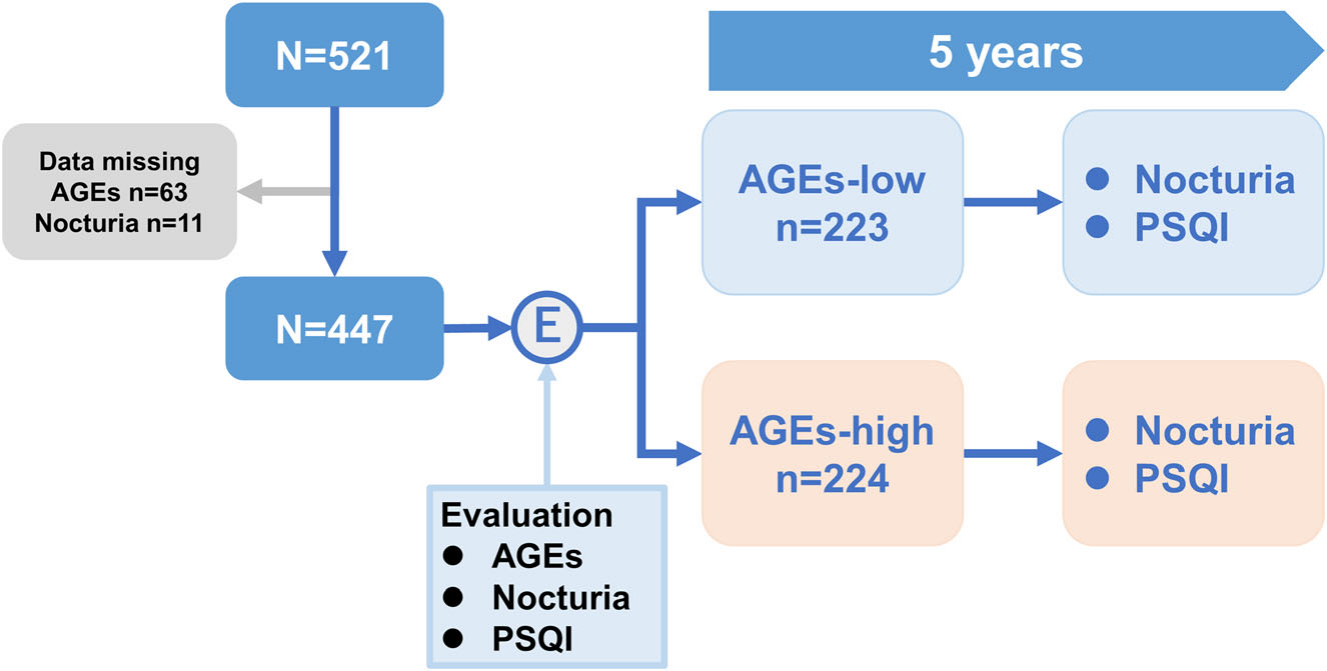
Selection of participants and stratification for AGEs‐low and ‐high groups. This study longitudinally evaluated the AGEs level, sleep disorders and nocturia frequency in 447 community‐dwelling adults for 5 years. Participants were divided into two groups: AGEs‐low (≤1.80) and AGEs‐high (>1.80). The longitudinal change of nocturia and PSQI for 5 years was compared between the AGEs‐low and AGEs‐high groups. AGEs, advanced glycation end products; PSQI, Pittsburgh Sleep Quality Index

**FIGURE 2:**
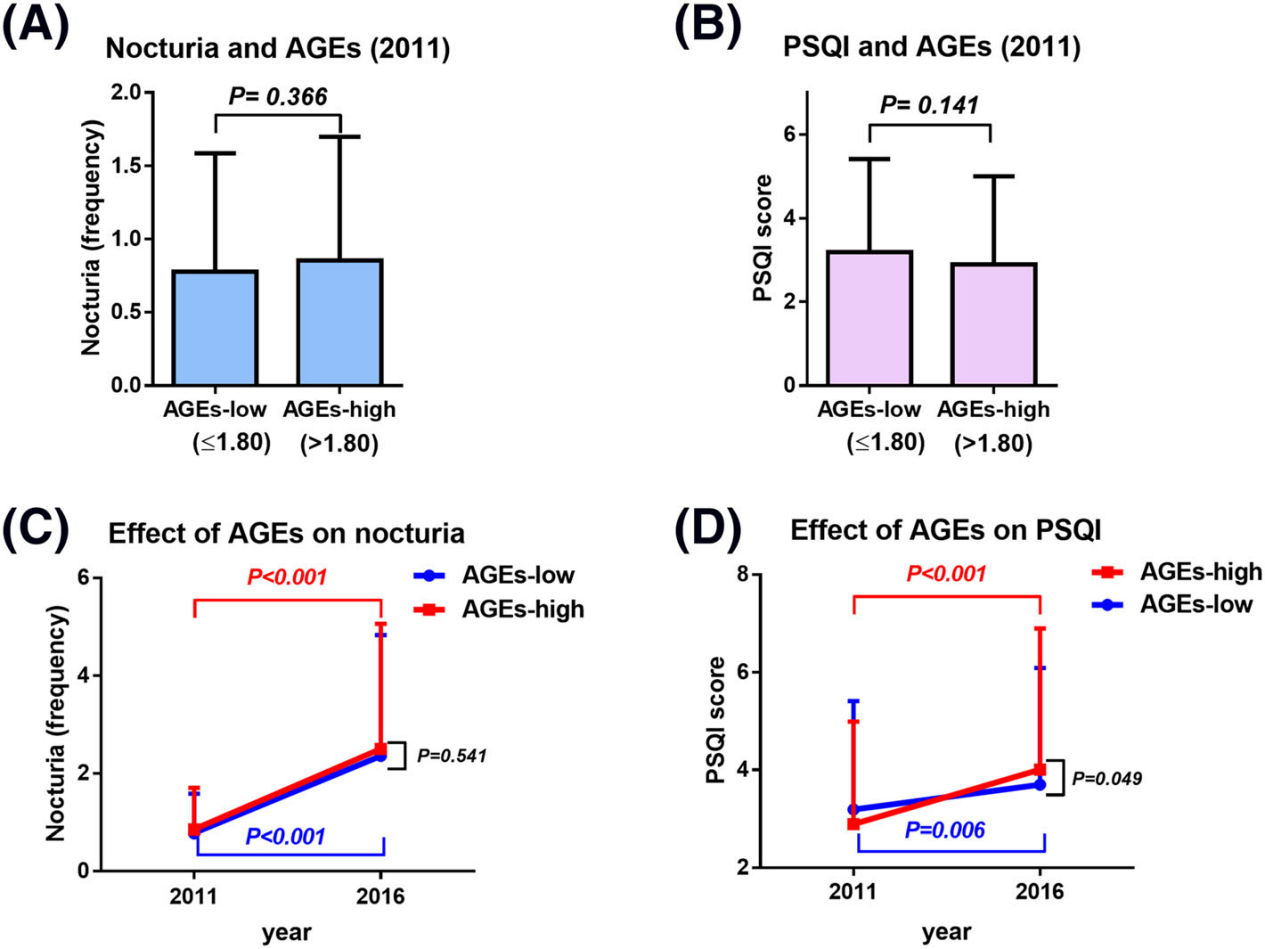
Comparison of nocturia or PSQI score from baseline to 5 years. Baseline nocturia frequency was compared between the AGEs‐low and AGEs‐high groups (A). Baseline PSQI score was compared between the AGEs‐low and AGEs‐high groups (B). The effect of baseline AGEs on worsening of nocturia (C) and worsening of PSQI score (D) was investigated 5 years later, respectively. AGEs, advanced glycation end products; PSQI, Pittsburgh Sleep Quality Index

Of the 521 participants, 74 with insufficient data such as skin AGEs (*n* = 63) and nocturia (*n* = 11) at baseline were excluded. Finally, 447 (170 were men and 277 were women) with a median age of 58 (IQR, 49–65) years were included in this study. The median frequency of nocturia and PSQI at baseline was 1 (IQR, 0–1) and 3 (1–4), respectively. The median value of skin AGEs was 1.799. Accordingly, the participants were divided into two groups according to the median value of skin AGEs: AGEs‐low (≤1.80, *n* = 223) and AGEs‐high (>1.80, *n* = 224; Figure 1). The background of the participants is presented in Table 1. A significant difference in the baseline age, gender and presence of DM exists between the AGEs‐low and AGEs‐high groups. However, there was no significant difference in nocturia frequency (Figure 2A) and PSQI score (Figure 2B) between the AGEs‐low and AGEs‐high groups at baseline.

**TABLE 1:** Background of participants

	All	AGEs‐low	AGEs‐high	*P* value
*n*	447	224	223	
Age, median (IQR)	58 (49–65)	57 (49–64)	59 (51–67)	*0.040*
Male, *n* (%)	170 (38%)	65 (29%)	105 (47%)	*<0.001*
BMI (kg/m^2^)	23 (21–25)	23 (20–26)	23 (21–25)	*0.220*
Somatic fat rate, %	26 (20–31)	27 (13–31)	25 (20–31)	*0.193*
Hypertension (HTN)	252 (56%)	127 (57%)	125 (56%)	*0.891*
Cardiovascular disease (CVD)	30 (6.7%)	10 (4.5%)	20 (8.9%)	*0.087*
Diabetes mellitus (DM)	41 (56%)	13 (5.8%)	28 (13%)	*0.021*
Sleeping tablet user, *n*	18 (4.0%)	6 (2.7%)	12 (5.4%)	*0.228*
IPSS	2 (1–6)	2 (1–5)	2 (1–6)	*0.950*
Nocturia > 1, *n*	70 (16%)	33 (15%)	37 (17%)	*0.603*
PSQI score > 5, *n*	62 (14%)	34 (15%)	28 (13%)	*0.412*
AGEs, median (IQR)	1.80 (1.34–2.21)	1.34 (1.32–1.55)	2.21 (1.97–2.53)	

Abbreviations: AGEs, advanced glycation end products; IQR, interquartile range, IPSS, International Prostate Symptom Score; PSQI, Pittsburgh Sleep Quality Index.

### 3.2 Longitudinal change of nocturia and PSQI for 5 years between the AGEs‐low and AGEs‐high groups

The nocturia frequency (Figure 2C) and PSQI score (Figure 2D) increased significantly for 5 years in both groups. However, the PSQI score increased significantly in the AGEs‐high group compared with the AGEs‐low group (Figure 2D; *P* = 0.049), although there was no difference in nocturia (Figure 2C; *P* = 0.541).

The effect of baseline AGEs on worsening of nocturia or PSQI score after 5‐year follow‐up.

**FIGURE 3:**
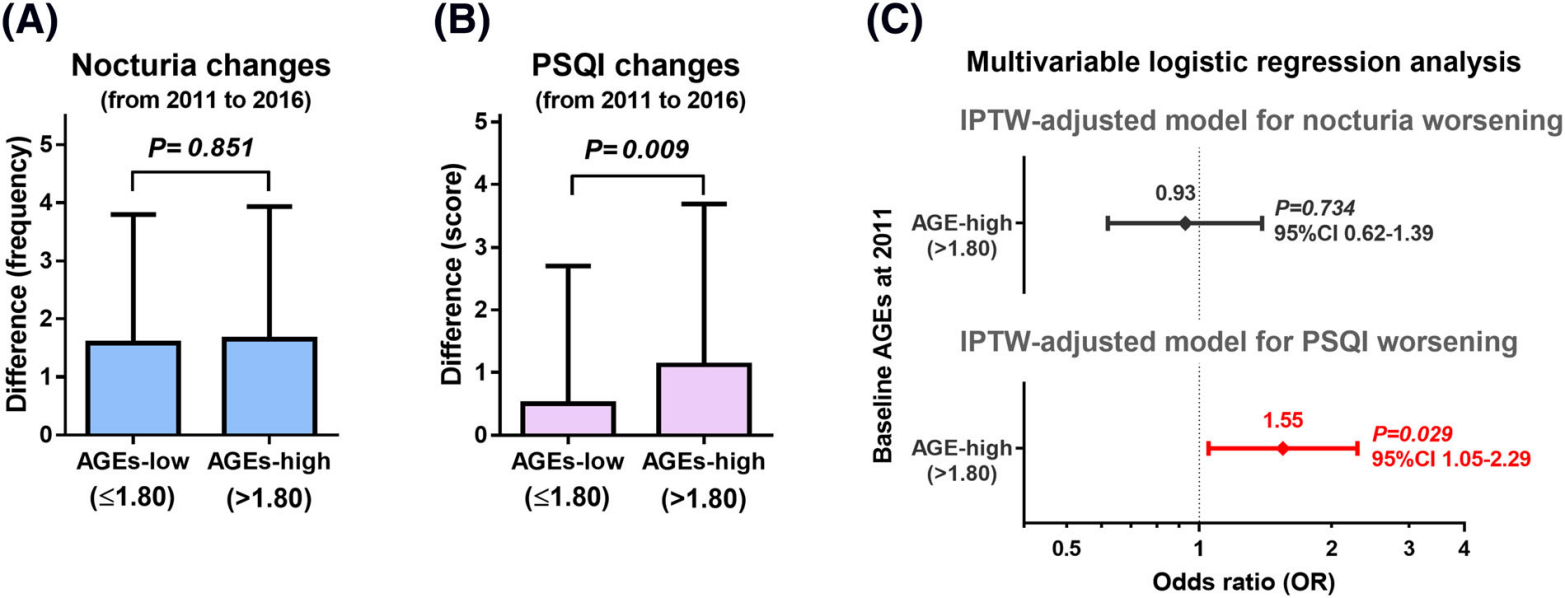
The effect of baseline AGEs on worsening of nocturia or PSQI score. Longitudinal change of nocturia for 5 years was compared between the AGEs‐low and AGEs‐high groups (A). Longitudinal change of PSQI score for 5 years was compared between the AGEs‐low and ‐high groups (B). The effect of baseline AGEs on worsening of nocturia or PSQI score was evaluated using background‐adjusted multivariable logistic regression analysis via the IPTW model (C). AGEs, advanced glycation end products; IPTW, inverse probability of a treatment weighting; PSQI, Pittsburgh Sleep Quality Index

The 5‐year difference of nocturia frequency was not significantly different between the AGEs‐low and AGEs‐high groups (Figure 3A). The 5‐year difference of PSQI score was significantly different between the AGEs‐low and AGEs‐high groups (Figure 3B). Multivariable IPTW‐adjusted logistic regression analysis showed a significant association between the AGE‐high and PSQI score worsening (*P* = 0.029; OR, 1.55; 95% CI, 1.05–2.29), whereas it was not the case in nocturia (*P* = 0.734; OR, 0.93; 95% CI, 0.62–1.39; Figure 3C).

## 4 DISCUSSION

Oxidative stress is well‐known to be involved in the pathogenesis of lifestyle‐related diseases, including atherosclerosis, HTN, DM, CVD and malignancies.^16^, ^17^ However, the association of oxidative stress with nocturia or sleep disorder remains unelucidated. This is the first study evaluating oxidative stress on the worsening of sleep disorders in a longitudinal analysis. This study demonstrated that AGEs had a significant impact on sleep disorder in community‐dwelling adults. Accumulated oxidative stress may play a key role in preceding sleep disorders, although the reason for the higher value of oxidative stress at baseline could not be addressed. Conversely, higher oxidative stress was not significantly associated with the worsening of the frequency of nocturia. It was an unexpected result because the positive association of oxidative stress (skin AGEs) on the frequency of nocturia >1 was previously reported.^17^ However, higher AGEs had limited effect for worsening of nocturia for 5 years. Taking these results together, the effect of oxidative stress may be different between sleep disorder and nocturia. This complexity may be related to whether nocturia is the main cause of awakening (related to urinary urgency) or awakening because of sleep disorders (unrelated to urinary urgency). ‘Nocturia is a result of sleep disorder because of high oxidative stress’ can be speculated because several previous studies suggested that sleep problems may be a key factor for preceding nocturia.^13^, ^14^, ^15^, ^28^ Although this connection could not be directly proven, the results may support the concept that nocturia is one of the consequences of the ageing syndrome (e.g., comorbidities and higher AGEs). However, there is not enough evidence to address this point. Thus, further studies are necessary to elucidate the complex association among oxidative stress, sleep disorders and nocturia.

The effect of sleeping tablets on nocturia or sleep disorder needs to be debated. This study identified 18 participants who used a sleeping tablet at baseline. However, there was no significant difference in the 5‐year differences of frequency of nocturia (*P* = 0.570; Figure S1A) and PSQI score (*P* = 0.598; Figure S1B) between the AGEs‐low and AGEs‐high groups. The use of sleeping tablets may have a limited effect on the improvement of nocturia or sleep disorder. However, clinical implication needs to be interpreted with caution because the duration of sleeping tablet use from this study could not be addressed. Sleep disorder treatment may be an option to improve nocturia, as several studies suggested.^13^, ^28^, ^29^, ^30^ A prospective multicentre study showed that ramelteon, a melatonin receptor agonist, had the potential to improve nocturnal voiding volume and nocturnal bladder capacity.^29^ Also, subsequent effect of ‘imidafenacin’ on sleep disorders in patients with overactive bladder was reported.^30^ However, the effect of those medications on oxidative stress remains unclear. Furthermore, sleeping medications are not a first‐line agent for nocturia treatment due to concerns on safety, especially for falls and fractures. Therefore, further studies are necessary to determine whether sleep problems or nocturia can improve oxidative stress.

The effect of OSAS on nocturia and oxidative stress needs to be discussed. OSAS is a chronic respiratory disorder that is closely related to sleep fragmentation and nocturia.^31^ Also, OSAS increases systematic oxidative stress via intermittent hypoxemia and consequent reoxygenation, which resulted in the production of reactive oxygen species.^32^ A recent study reported that the prevalence of nocturia in patients diagnosed with OSAS is very high (75.8%) in men and women. Also, they reported that almost half of patients experienced a decrease in the nocturia frequency after treatment with continuous positive airways pressure.^31^ These results suggested that OSAS has a key role in oxidative stress and nocturia. However, there are complex pathophysiological mechanisms between nocturia and sleep disorders. Because both nocturia and sleep disorders have been implicated in oxidative stress, the role of oxidative stress in sleep physiology and sleep disorders may be key factors for future nocturia researches.^3^

Limitations include the small sample size, selection bias of age and gender, limited number of evaluations, reliance on self‐reported measures and other unmeasurable confounding factors that cannot be controlled. Moreover, this study could not address the number of patients with OSAS and nocturnal polyuria in this cohort because of the lack of information. Furthermore, this study had no data for the concomitant use of an anticholinergic agent and selective β3‐adrenoceptor agonist to treat overactive bladder. Also, we do not have information on the types and dosage of sleeping medication. Finally, the results of this study may not be generalizable to other populations. Despite these limitations, this is the first longitudinal study evaluating the effect of oxidative stress on the worsening of sleep disorder or nocturia. The current results emphasize the importance of recognizing the effect of oxidative stress on sleep problems and nocturia in community‐dwelling adults.

In conclusion, this longitudinal study demonstrated the significant effect of oxidative stress on worsening sleep disorders in community‐dwelling adults. However, oxidative stress may not significantly worsen the nocturia frequency.

## CONFLICT OF INTEREST

All authors have declared no conflicts of interests.

## FUNDING INFORMATION

This work was supported by the Japan Society for the Promotion of Science (JSPS) KAKENHI (Grant 19H05556 and 20K09517) and by the Japan Science and Technology Agency, Center of Innovation Program (JPMJCE1302).

## AUTHORS' CONTRIBUTION

Data collection, manuscript writing: Sakae Konishi. Project development, data collection, manuscript writing, project management: Shingo Hatakeyama. Data collection, project management: Atsushi Imai. Data collection: Kazutaka Okita. Data collection: Koichi Kido. Data collection: Yusuke Ozaki. Data collection, project management: Nozomi Uemura. Data collection, project management: Takuro Iwane. Data collection, project management: Teppei Okamoto. Data collection: Hayato Yamamoto. Data collection: Takahiro Yoneyama. Data collection: Yasuhiro Hashimoto. Project management: Chikara Ohyama.

## ETHICS STATEMENT

The study was conducted in accordance with the ethical standards of the Declaration of Helsinki. This study was approved by the Ethics Committee of Hirosaki University School of Medicine (authorization number, 2014–015, 2019–099).

## INFORMED CONSENT

All subjects provided written informed consent before participating in the study.

## Supporting information


**Figure S1.** Supplemental figure: longitudinal change of nocturia or PSQI score for 5 years in participants using sleeping tablet
Longitudinal change of nocturia for 5 years was compared between the AGEs‐low and AGEs‐high groups (**A**). The longitudinal change of PSQI score for 5 years was compared between the AGEs‐low and AGEs‐high groups (**B**).
