# Supplementary figures and images for: Effect of advanced glycation end products on nocturia or sleep disorders: A longitudinal study
Source: BJUI Compass. 2021 Oct 5;3(2):162–8. doi: 10.1002/bco2.114 (PMC8988819; doi:10.1002/bco2.114)

Fig.S1

A

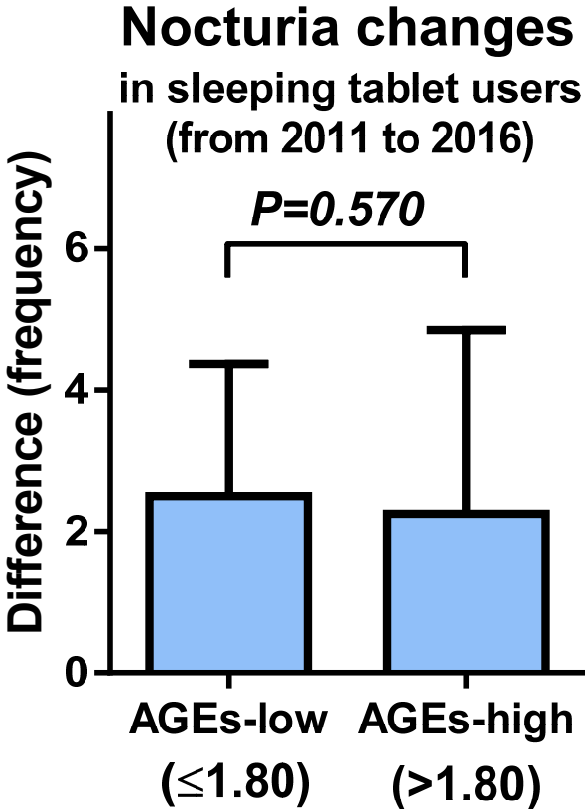

B

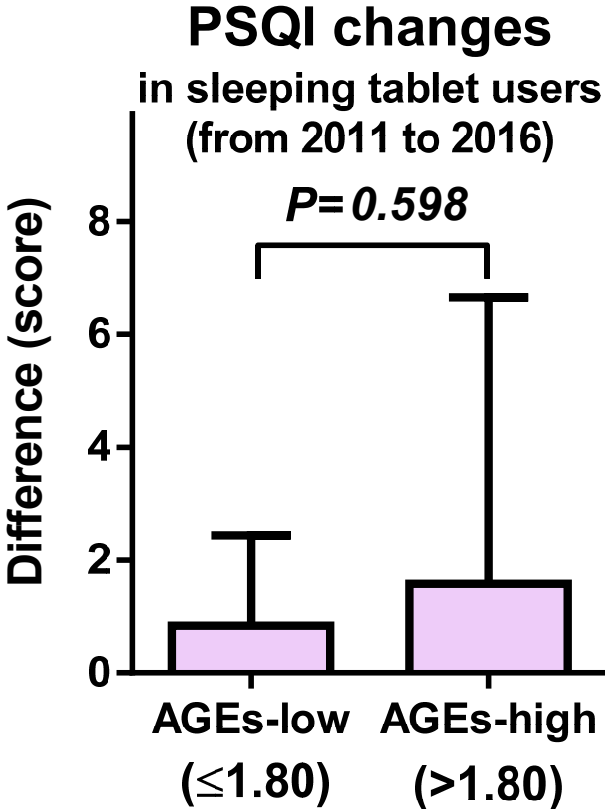

Supplement: Supplementary file 1 — Figure S1. Supplemental figure: longitudinal change of nocturia or PSQI score for 5 years in participants using sleeping tablet Longitudinal change of nocturia for 5 years was compared between the AGEs‐low and AGEs‐high groups (A). The longitudinal change of PSQI score for 5 years was compared between the AGEs‐low and AGEs‐high groups (B). [file BCO2-3-162-s001.pdf]
